# Characterization of a Natural, Stable, Reversible and Colourful Anthocyanidin Network from *Sphagnum* Moss Based Mainly on the Yellow *Trans*-Chalcone and Red Flavylium Cation Forms

**DOI:** 10.3390/molecules26030709

**Published:** 2021-01-29

**Authors:** Helge Berland, Øyvind M. Andersen

**Affiliations:** Department of Chemistry, University of Bergen, P.O. 7803, N-5020 Bergen, Norway

**Keywords:** 3-deoxyanthocyanidin, A–E rings, *trans*-chalcone, structure elucidation, equilibrium network, colours, NMR

## Abstract

Anthocyanins with various functions in nature are one of the most important sources of colours in plants. They are based on anthocyanidins or 3-deoxyanthocyanidins having in common a C15-skeleton and are unique in terms of how each anthocyanidin is involved in a network of equilibria between different forms exhibiting their own properties including colour. Sphagnorubin C (**1**) isolated from the cell wall of peat moss (*Sphagnum* sp.) was in fairly acidic and neutral dimethyl sulfoxide characterized by nuclear magnetic resonance (NMR) and ultraviolet–visible (UV–vis) absorption techniques. At equilibrium, the network of **1** behaved as a two–component colour system involving the reddish flavylium cationic and the yellow *trans*–chalcone forms. The additional D- and E-rings connected to the common C15-skeleton extend the *π*-conjugation within the molecule and provide both bathochromic shifts in the absorption spectra of the various forms as well as a low isomerization barrier between the *cis-* and *trans-*chalcone forms. The hemiketal and *cis-*chalcone forms were thus not observed experimentally by NMR due to their short lives. The stable, reversible network of **1** with good colour contrast between its two components has previously not been reported for other natural anthocyanins and might thus have potential in future photochromic systems. This is the first full structural characterization of any naturally occurring anthocyanin chalcone form.

## 1. Introduction

Anthocyanins are ubiquitous in plants, and the total number of anthocyanin structures identified after isolation from plant extracts is about 800. These pigments are mainly divided into two groups, anthocyanins and 3-deoxyanthocyanins, with the latter group lacking an oxygen-function on the anthocyanidin 3-position on the C-ring [1]. The vast majority of the common anthocyanins are based on just 6 different aglycones (anthocyanidins) varying with the substitutions on the B-ring, and range in colour from salmon red to dark purple and blue [2]. In addition, a third heterogenous group (pyranoanthocyanins) [3,4] formed from anthocyanins during storage and processing in plant-derived foods, has gained much attention mostly because of their influence on colour evolution in wine during maturation [5]. A couple of pyranoanthocyanins have also been reported to occur in minor amounts in intact plant materials, for instance strawberries [6]. The various pyranoanthocyanins have in common an extra pyranic ring formed by cyclic addition onto both carbon 4 and the hydroxyl group at carbon 5 of the anthocyanidin skeleton. In solution, each anthocyanin exists in a network of reactions between the flavylium cation, hemiketals, chalcones and quinoidal bases [7,8,9]. The vast majority of structure elucidations of anthocyanins have been undertaken in acidic environments on the flavylium cation form, which is considered to be the most stable form. In recent years it has been shown that synthetic flavylium compounds analogous to anthocyanidins also possess the same general network of chemical reactions [10].

In this context, pigment **1**, Sphagnorubin C isolated from sphagnum moss, is an interesting molecule belonging to the 3-deoxyanthocyanidin group. However, due to the two extraordinary aromatic rings connected to the A-ring, this pigment possesses properties not previously highlighted for any natural anthocyanidin/anthocyanin before. The purpose of this work was to present the first full structural characterization of a naturally occurring anthocyanidin/anthocyanin chalcone form. Other aims were to focus on how the extra aromatic rings of **1** extend the type of network of equilibrium forms displayed by natural anthocyanins, and to reveal the colours of the different forms of **1** in this network and their potential use.

## 2. Results

### 2.1. Structural Elucidation

The structures of the different forms of **1** present in deuterated dimethyl sulfoxide (*d*-DMSO), with and without deuterated trifluoroacetic acid (*d*-TFA), were elucidated using a combination of 1D (^1^H) and 2D (heteronuclear single quantum coherence (HSQC), heteronuclear multiple bond coherence (HMBC), double-quantum filtered correlation spectroscopy (DQF-COSY)) NMR experiments and high-resolution electrospray ionisation mass spectrometry (HR-ESI-MS).

#### 2.1.1. Elucidation of the Flavylium Cationic Form of **1**

**Figure 1:**
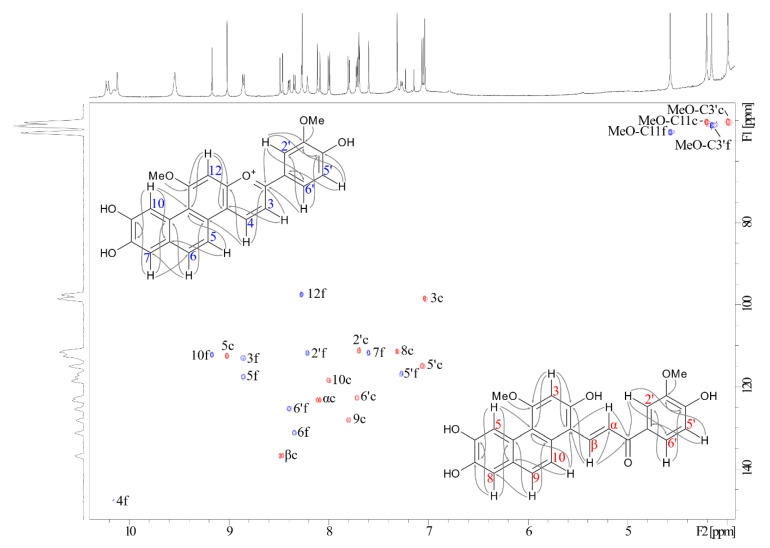
Heteronuclear single quantum coherence (HSQC) nuclear magnetic resonance (NMR) spectrum of Sphagnorubin C (**1**) in pure deuterated dimethyl sulfoxide (*d*-DMSO) after equilibration for 24 h, showing the ^1^H-^13^C cross-peaks of both the flavylium cation (blue) and the *trans*-chalcone (red) forms. The ^1^H-NMR spectrum and the ^13^C-NMR projection of **1** are aligned on the top and left axes, respectively. The flavylium cation (blue numbers) and *trans*-chalcone (red numbers) structures are supplied with arrows showing long-range ^1^H-^13^C heteronuclear multiple bond coherence (HMBC) cross-peaks. The flavylium cation HMBC cross-peaks are collected from a sample of **1** dissolved in 5% deuterated trifluoroacetic acid (*d*-TFA) in *d*-DMSO.

The ^1^H spectrum of **1** dissolved in 5% *d*-TFA in *d*-DMSO (*v*/*v*) showed an aromatic region corresponding to 10 proton signals (see supplementary Figure S1). Three of these protons belonged to an AMX coupling pattern at *δ* 8.09 ppm (d, 2.2 Hz; H2’), *δ* 7.16 ppm (d, 8.6 Hz; H5’) and *δ* 8.26 ppm (dd, 2.2, 8.6 Hz; H6’), which together with one methoxy group at *δ* 4.03 ppm coupled to C3’ shown through cross-peaks in the HMBC spectrum (Supplementary Figure S2), were similar to the corresponding signals of the B-ring of the anthocyanidin peonidin [11]. The rest of the proton and carbon atoms of the B-ring were assigned (Table 1) based on the HMBC and HSQC spectra (Supplementary Figure S3). The aromatic region also contained a signal with a rather downfield chemical shift (10.04 ppm) typical for anthocyanidin H4 protons. This signal having a coupling constant of 9.1 Hz, was shown by the DQF-COSY spectrum (Supplementary Figure S4) to be coupled to a proton at *δ* 8.74 ppm (H3). Both H4 and H3 as well as H2’ had cross-peaks with C2 (*δ* 168.98 ppm) in the HMBC spectrum, supporting the assignment of a 3-deoxyanthocyanidin C-ring. The rest of the carbon atoms of this ring was assigned by the HMBC spectrum. In this spectrum the proton at *δ* 8.13 ppm (H12) has in common with H4 a cross-peak with C12a (*δ* 158.35 ppm), and in common with H3 a cross-peak with C4a (*δ* 117.81 ppm). The H12 proton also helped to assign the second methoxy group to C11, by its cross-peaks with this carbon (*δ* 168.68 ppm) (Figure 1). The remaining carbon atoms of the A-ring, C10b and C4b, were assigned by cross-peaks with H12 (*δ* 8.13/119.70 ppm) and H4 (*δ* 10.04/129.43 ppm), respectively.

The elucidation of the unusual D- and E-rings started with assignment of H5 (*δ* 8.72 ppm, d 9.0 Hz) having in common with H12 (*δ* 8.13 ppm, s) a cross-peak to C10b (*δ* 8.72/119.70 ppm) in the HMBC spectrum (Figure 1). H5 showed in the DQF-COSY spectrum cross-peak at *δ* 8.72/8.20 ppm, used for assignment of H6. This latter proton had in common with H4 cross-peak to C4b (*δ* 8.20/129.43 ppm), and in common with H7 (*δ* 7.47 ppm, s) cross-peak to C10a (*δ* 8.20/123.77 ppm) in the HMBC spectrum. The last carbon of the D-ring, C6a, was assigned by the cross-peak at *δ* 8.72/129.27 ppm (H5/C6a). This carbon also showed a cross-peak to H10 of the E-ring (*δ* 9.05/129.27 ppm). H10 had another ^3^J_HC_ cross-peak to C8 (*δ* 9.05/147.57 ppm). The assignments of the remaining C-atom of E-ring, C9, was revealed by the ^3^J_HC_ cross-peak between H7 and C9 (*δ* 7.47/147.99 ppm). With all the proton signals assigned, the HSQC spectrum confirmed the chemical shifts of the corresponding tertiary carbon atoms (Figure 1). The positive mode HR-ESI-MS spectrum of **1** in acidified DMSO showed a molecular ion at *m/z* 415.11833 corresponding to the empirical formula C_25_H_19_O_6_^+^ (calc. *m/z* 415.11816), which is in agreement with **1** in its flavylium cationic form. See supplementary Figure S7 for the MS spectrum.

#### 2.1.2. Elucidation of the *Trans*-Chalcone Form of **1**

The ^1^H spectrum of **1** dissolved in pure *d*-DMSO and equilibrated at room temperature for 24 h showed, in addition to signals of the flavylium cationic form, a set of peaks corresponding to another form of **1**. Resonances of three of these protons (H2’, H5’ and H6’) had a similar AMX coupling pattern as elucidated for the B-ring of the flavylium form (Table 1). The carbon resonances of this ring were determined by the cross-peaks in the HSQC and HMBC spectra of this sample (Figure 1). Interestingly, both H2’ and H6’ had cross-peaks in the HMBC spectrum to the same carbon at *δ* 187.78 ppm corresponding to a carbonyl function. This carbonyl group also showed cross-peaks to two protons (Hα and Hβ) (Figure 1), which coupled with each other with a large coupling constant (15.4 Hz) typical for a *trans*-alkene. The Hα proton also had a cross-peak to C1 (*δ* 7.97/108.77 ppm), while Hβ also showed cross-peaks to C1 (*δ* 8.34/108.77 ppm) and C2 (*δ* 8.34/156.38 ppm), which were in accordance with an ‘open C-ring’ of a chalcone form. The ^1^H-^1^H coupling patterns of the five remaining protons of this second form of **1** in the proton and DQF-COSY spectra were similar to the corresponding patterns of the analogous protons of the A-, D- and E-rings of the flavylium cationic form of **1**. See supplementary Figure S5 for the DQF-COSY spectrum. As described above for the flavylium cationic form, the tertiary carbon atoms of the A-, D- and E-rings of the second form were assigned from cross-peaks in the HSQC spectrum (see supplementary Figure S6) after cross-referencing to cross-peaks in the HMBC spectrum (Figure 1). After assignments of the remaining quaternary carbon atoms of these rings (Table 1) by the cross-peaks in the HMBC spectrum, the second form of **1** was elucidated to be the *trans*-chalcone form of **1**. The negative mode HR-ESI-MS spectrum of a sample of this *trans*-chalcone form showed a molecular ion at *m/z* 431.11338 corresponding to the empirical formula C_25_H_19_O_7_^-^ (calc. *m/z* 431.11308), in agreement with a deprotonated *trans*-chalcone form of **1**. See supplementary Figure S8 for the MS spectrum.

### 2.2. The Network of Anthocyanin Forms

**Scheme 1:**
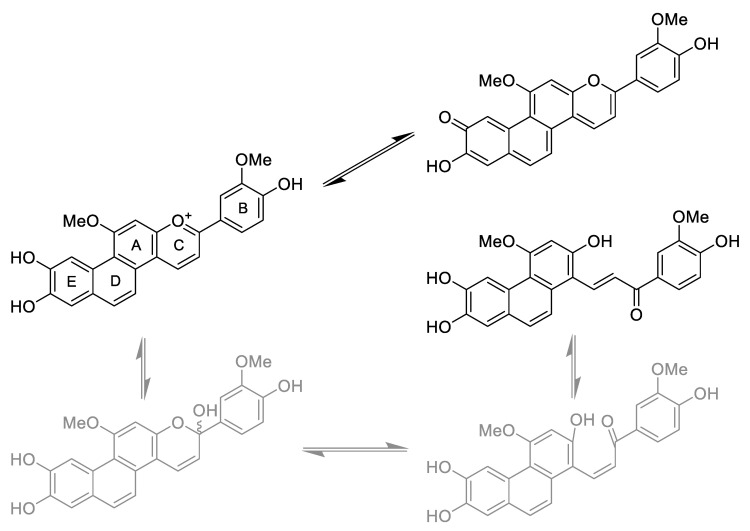
The hemiketal and *cis-*chalcone forms were short-lived and not experimentally detected (represented in the figure with faded structures). The quinoidal base in the figure represents various structures differing in the position of the carbonyl function.

The red sample of **1** dissolved in 5% *d*-TFA in *d*-DMSO (*v*/*v*) (sample A), showed in the NMR spectra only the presence of signals corresponding to the flavylium cation form, also during storage for 88 days at −20 °C. When **1** was dissolved in pure *d*-DMSO (sample B), the first ^1^H spectrum recorded after 30 min also revealed the presence of just the flavylium cationic form. However, by regular recordings of ^1^H spectra throughout 24 h at room temperature another set of peaks belonging to the characterised *trans*-chalcone form, were recognized. During this period the flavylium cation and *trans*-chalcone forms were stabilised at a 1:1 equilibrium ratio, and the colour of the sample had turned orange. No hemiketal or *cis*-chalcone forms nor any degradation products were detected in the NMR spectra. After storage for 76 days at −20 °C, sample B was acidified with 5% *d*-TFA. Following 1 h of storage of this ‘new’ sample B at room temperature, the NMR spectra still revealed an approximate 1:1 equilibrium ratio of the *trans*-chalcone and flavylium forms without distinctive signs of other compounds. However, after 6 days in a frozen solvent at −20 °C the flavylium cation and only minor amounts of the *trans*-chalcone form occurred in the sample, and this latter form was completely gone after 4 more days. This network consisting of the pure reddish flavylium cation and the orange coloured flavylium cation/*trans*-chalcone mixture (1:1) as endpoints (Scheme 1), thus seemed to be both fully stable and reversible under the changing pH conditions described above, without any signs of plausible hemiketal or *cis*-chalcone forms nor any degradation products. 

**Figure 2:**
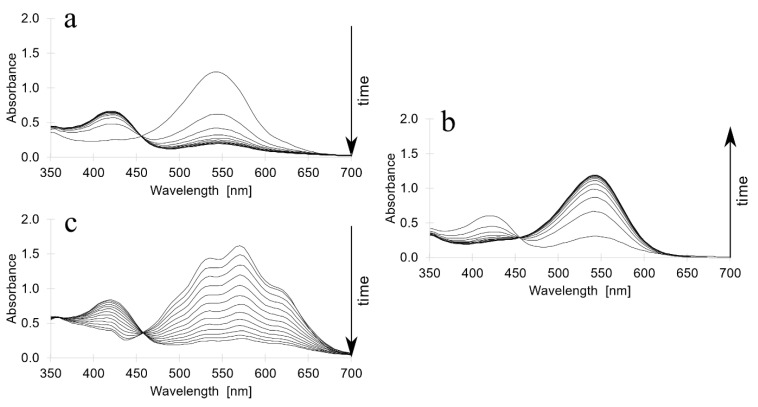
Visible absorption spectra of samples of Sphagnorubin C (**1**) recorded at 2-h intervals for 24 h, with different pre-treatments of the samples (see Section 4.2). (**a**) Sample D dissolved in pure DMSO: The spectra show the decrease of the flavylium cation form (*λ*_max_ at 540 nm) and the increase of the *trans*-chalcone form (*λ*_max_ at 420 nm) with time. Equilibration is obtained after circa 14 h. (**b**) Sample E (Sample D after 24 h acidified with 5% TFA): The spectra show the increase of the flavylium cation form and the decrease of the *trans*-chalcone form with time. (**c**) Sample F_3_ (see Section 4.2) dissolved in pure *d*-DMSO: The spectra show the decrease of the quinoidal forms and the increase of the *trans*-chalcone form with time. F_3_ has initially a dark blue/purple colour typical for quinoidal forms. 5% TFA was also added to sample F_3_ after 24 h, which gave virtually identical results to those described for sample E, Note the isosbestic point at 455 nm in all the three spectra series. The spectra were recorded at room temperature.

The colours of the network of various anthocyanidin forms of **1** were also studied using visible absorption spectroscopy having faster sampling time and less demand for sample amounts per experiment compared to the NMR experiments. To reduce possible effects of residual water and acid coming from the isolation process, the bulk sample from where samples A and B originated was treated according to Section 4.2 to give samples C–F. Sample C (**1** dissolved in 0.5% TFA in DMSO) had the characteristic red colour of the flavylium cationic form with a *λ*_max_ around 540 nm in its visible absorption spectrum. When **1** was dissolved in pure DMSO (sample D), a relatively slow transformation from red, though orange, to yellow was observed. The yellow solution had its *λ*_max_ around 420 nm (Figure 2a), in accordance with the transformation from the red flavylium cationic form into the yellow *trans*-chalcone form observed in the NMR experiments described above. However, contrary to sample B, sample D showed an almost complete conversion from the flavylium cation form to the *trans*-chalcone form (reasoned by the low absorbance of the flavylium cation form at 540 nm, Figure 2a) reaching equilibrium after circa 14 h. After another 24 h, sample D was acidified with 5% TFA (*v*/*v*) (sample E). Similarly, as observed in the NMR experiments described above, a slow transformation of the *trans*-chalcone form back to the flavylium cationic form was recognized by colour change from yellow to red, reaching equilibrium after circa 16 h (Figure 2b). After another 24 h period, the flavylium cation absorbance of sample E was at 97% of its initial absorbance at 540 nm in sample D. The 3% reduction in absorbance at 540 nm in sample E was in accordance with the volume of the added acid to sample D. The flavylium cationic form thus seemed to be fully regenerated from the *trans*-chalcone form, without any sign of degradation. 

**Figure 3:**
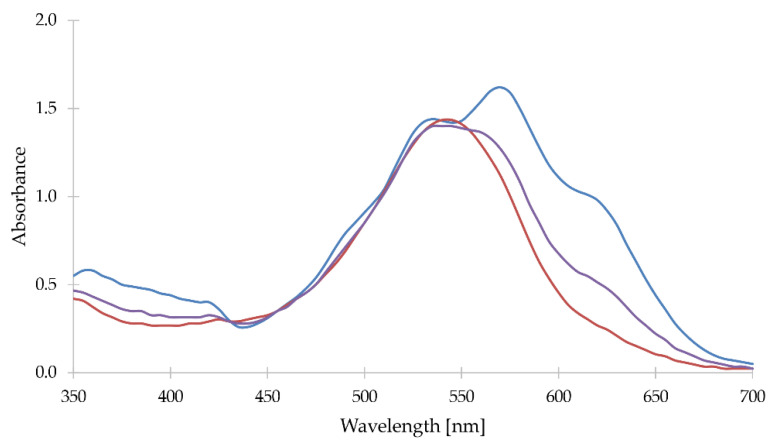
Visible absorption spectra of samples F_1_ (red), F_2_ (purple) and F_3_ (blue) of Sphagnorubin C (**1**). The samples, which have different pre-treatment (see Section 4.2), were dissolved in pure DMSO/*d*-DMSO. The spectra show that different amounts of the flavylium cationic form were transformed into quinoidal forms depending on the pre-treatment of each of the samples. The spectra are normalised to the isosbestic point at 455 nm.

A typical anthocyanidin network also includes quinoidal structures, which are formed in simple acid-base reactions starting with the flavylium cation under near neutral to basic pH conditions [7]. The importance of the impact of minor amounts of residual water/acid in the pigment **1** sample (here sample F) on such acid-base reactions is visualized in Figure 3. The red line represents the first recorded visible absorption spectrum of sample F_1_ purified at the same stage as sample D (see Section 4.2). Here sample F_1_ has its *λ*_max_ at 540 nm, in accordance with the flavylium cationic form. The purple and blue lines in represent the first recorded visible absorption spectrum of samples F_2_ and F_3_, respectively. Samples F_2_ and F_3_ were prepared by adding respectively one and two purification steps in addition to those used for preparation of F_1_. Each of these purification steps included adding pure methanol to the pigment sample followed by evaporation to dryness under a stream of nitrogen before pure DMSO (*d*-DMSO for F_3_) finally was added as solvent. In these additional steps minor amounts of residual water/acid was removed from the sample, and the pigment obtained its neutral form. Sample F_3_ gave initially a dark blue/purple colour when dissolved in pure deuterated DMSO. The initial visible absorption spectrum of this sample (blue line in Figure 3) having its *λ*_max_ at 570 nm and additional shoulders above 600 nm, was in accordance with quinoidal forms of **1**. The initial visible absorption spectrum of sample F_2_ (purple line in Figure 3) showed an intermediate spectrum between the spectra of F_1_ and F_3_, respectively, in accordance with a mixture of flavylium cation and quinoidal forms of **1**. Visible absorption spectra of sample F_3_ were also recorded every 2 h for 24 h (Figure 2c), which also showed the slow transformation from the quinoidal forms to the *trans*-chalcone. These observations were in accordance with the formation of the flavylium cationic form from the quinoidal forms before the reactions towards the thermodynamically more stable *trans*-chalcone started (Scheme 1), as described above for sample D.

## 3. Discussion

The structure of Sphagnorubin C (**1**) in its flavylium cation form has previously been elucidated using derivatisation techniques in combination with ultraviolet–visible (UV–Vis), infrared (IR) and ^1^H spectroscopy and mass spectrometry [12,13]. In a comparison of the assignments of ^1^H shift values verified for **1** in its flavylium cation form in the same solvent (DMSO), complete assignments of H2’ and H6’ and a switch between H12 and H7 were the only differences, which in the present study were determined by 2D NMR techniques. 

Pigment **1** has previously not been supplied with ^13^C-NMR data. However, highly relevant for the present paper, the 3’-OH analogue (Sphagnorubin B) of **1** has previously been made into a pentaacetate-chalcone derivative in the slightly alkaline solvent when reacted with ethanoic anhydride and pyridine [13]. This derivative was supplied with ^13^C-NMR data, although many of the signals were not completely assigned.

Anthocyanidins and anthocyanins (anthocyanidin with glycosidic moieties) are outstanding compounds by virtue of the way they are involved in a series of equilibrium reactions giving rise to several forms (secondary structures). However, proper ^1^H and ^13^C-NMR structural assignments of other forms than the flavylium cationic form, have with just a couple of exceptions not been addressed for anthocyanins and anthocyanidins. As far as we know the first proper spectroscopic data (UV–visible absorption spectrum) for any natural anthocyanin in its chalcone form was published by Brouillard, et al. [14]. In 1993, Santos, et al. [15] studied various equilibrium forms of malvidin 3,5-*O*-diglucoside (malvin), which coexisted in slightly acidic aqueous conditions. Two-dimensional nuclear Overhauser effect spectroscopy (NOESY) exchange correlation NMR spectra provided valuable information about the structural transformations, including evidence for the relationship between the various equilibrium forms of malvin. For the first time the chalcone forms of a natural anthocyanin were characterized by assignments of the chemical shifts of the four protons of the anthocyanidin. Later the chemical proton shifts of the chalcone forms of malvidin 3-*O*-glucoside [16] and cyanidin 3-*O*-glucoside [17] have been assigned. Several forms of the pyranoanthocyanin vitisin A., including the chalcone form, have previously been reported to be distinguished by NMR [3]. However, the authors reported that complete unambiguous assignment of the ^13^C-NMR signals of the chalcone form was not possible. More recently it has clearly been demonstrated that pyranoanthocyanins like vitisins are not able to form hemiketals [18,19], and as a consequence unable to form chalcone forms. Thus, the assignments of all ^1^H and ^13^C-NMR signals of the *trans*-chalcone structure of **1** is as far as we know, the first full structural characterization of a naturally occurring anthocyanin chalcone form.

The difference in structure between anthocyanidins and 3-deoxyanthocyanidins is the lack of oxygen function in the 3-position in the C-ring of the latter group. The impact of a reduced chromophore gives the common 3-deoxyanthocyanins (apigeninidin, luteolinidin and tricetinidin) yellow colours under acidic conditions compared to more scarlet to blue colours of analogous flavylium cationic forms of the common anthocyanins. Another even more striking effect of this hypsochromic shift in the absorption spectra under less acidic/neutral conditions is displayed by the colourless chalcone forms of the common 3-deoxyanthocyanins contrasting the yellow chalcone forms of the analogous anthocyanins. However, the colours of 3-deoxyanthocyanidins are positively reckoned to be much more stable than the colours of the analogous anthocyanins under slightly acidic conditions [20]. The network consisting of the flavylium cationic and *trans*-chalcone forms of pigment **1** in the present paper, proved the best of both of these attributes. The additional D- and E-rings extended the *π*-conjugation within the molecule and provided bathochromic shifts in the absorption spectra of the various forms of **1** compared to 3-deoxyanthocyanidins comprising only the normal A–C rings. Thus, the red flavylium cationic form of **1** had its absorption maximum (*λ*_max_) at 540 nm, while the *trans*-chalcone form revealed a yellow colour and had its *λ*_max_ at 420 nm in pure DMSO.

**Figure 4:**
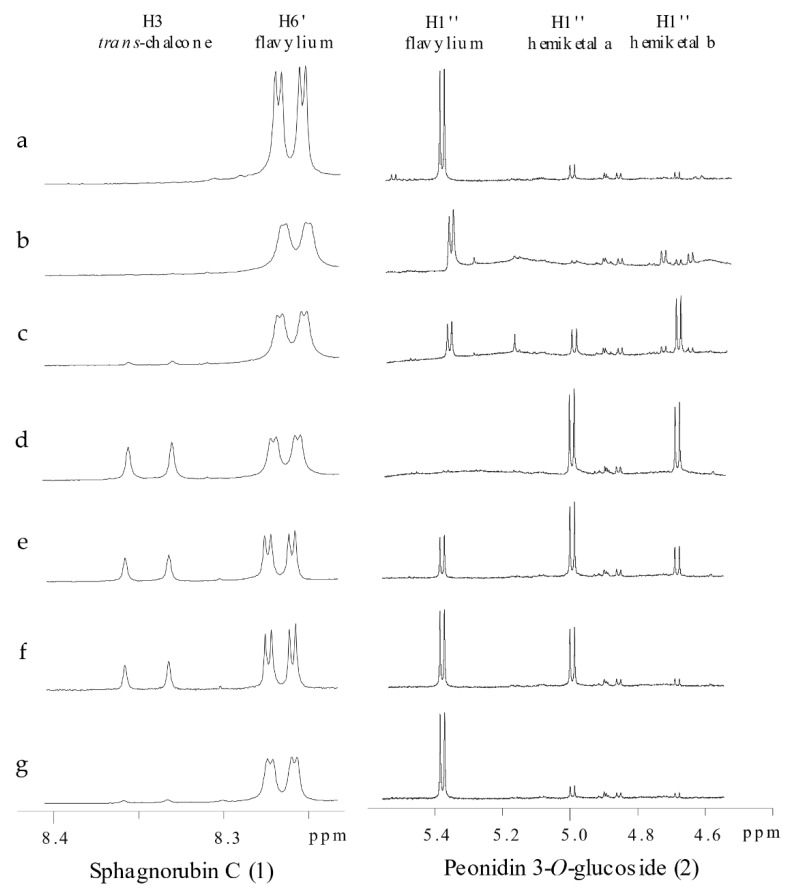
^1^H-NMR spectra of Sphagnorubin C (**1**, left) and peonidin 3-*O*-glucoside (**2**, right) showing selected signals of different equilibrium forms belonging to each of the pigments. The spectra are recorded when **1** and **2** are separately (**a**) dissolved in 5% *d*-TFA in *d*-DMSO, (**b**) dissolved in pure *d*-DMSO, (**c**) after samples **b** have been equilibrated for 1 h, (**d**) after samples **c** have been equilibrated for 3–7 days, (**e**) after samples **d** have been acidified with 5% *d*-TFA, (**f**) after samples **e** have been equilibrated for 1 h, (**g**) after samples **f** have been equilibrated for 5–6 days. The flavylium cation and *trans*-chalcone forms of **1** are represented by their H6’ and H3 protons, respectively, while both the flavylium cation and the two hemiketal enantiomers of **2** are represented by their anomeric H1’’ protons. The spectra were recorded at 21 °C.

**Scheme 2:**
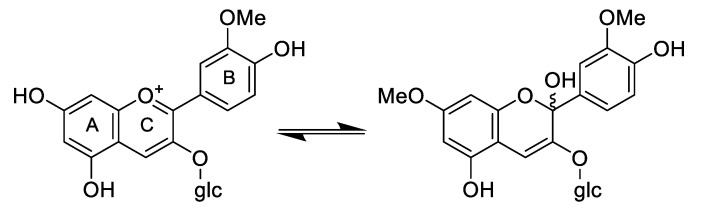
Peonidin 3-*O*-glucoside (**2**) dissolved in pure *d*-DMSO. After 24 h storage the two hemiketal enantiomers were present at a 56:44 ratio, and only traces of other potential equilibrium forms were revealed.

How was it verified that pigment **1** had higher colour stability than the common anthocyanins (in this paper represented by peonidin 3-*O*-glucoside, **2**) under slightly acidic to neutral conditions? The flavylium cation and the two hemiketal enantiomers of **2** were in the ^1^H NMR spectra in Figure 4 (right) represented by their anomeric protons (see Supplementary Table S1 for aromatic and anomeric ^1^H-NMR data). In pure *d*-DMSO the flavylium cationic form of **2** was transferred within hours into two hemiketal forms (64% within 1.4 h or 93% after 4.9 h) (Figure 4, right, a–d), according to Scheme 2. Few traces of the probable yellow chalcone forms were observed (Figure 4, right). This is in accordance with previous papers reporting the chalcone forms to constitute typically less than 10% of the various equilibrium forms of anthocyanins (not 3-deoxyanthocyanidins) in slightly acidic or neutral aqueous solutions [8]. The important factor in this context was that the hemiketal forms of **2** (or other anthocyanins) were colourless, making the colour of the solution fade proportionally with the increase of the hemiketal forms. However, opposite to this pattern the red flavylium cation and the yellow *trans*-chalcone forms occurred in various proportions when **1** was examined under similar conditions as described for **2** (Figure 4, left, a–d). The *trans*-chalcone and flavylium cationic forms in the ^1^H-NMR spectra are in Figure 4 (left) represented by their H3 and H6′ protons, respectively. Since no colourless forms of **1** were observed in the NMR spectra, a gradual change from reddish flavylium cation colour towards yellow *trans*-chalcone colours were observed in the NMR spectra of this sample taken during storage (Figure 4, left, a–d). To characterize the reversibility of the two-components system described above between the flavylium cation and *trans*-chalcone forms of **1**, acid (5% *d*-TFA, *v*/*v*) was added to the equilibrated neutral sample (Figure 4, left, e–f). After 5–6 days of equilibration, the sample had turned back to its red flavylium cationic form, leaving only traces of the *trans*-chalcone form (Figure 4, left, g).

It is well known that all anthocyanins, including 3-deoxyanthocyanidins, are represented by a network of chemical forms, which are reversibly interconverted by external stimuli such as pH variation, light, etc. [8]. Based on the results described above, the network depicted for **1** is presented in Scheme 1. It can be viewed as a single acid-base equilibrium involving the flavylium cation and the overall conjugated base. The conjugated base represents both the quinoidal bases, hemiketal forms and the *cis-* and *trans-*chalcones. The hemiketal and *cis-*chalcone forms are short-lived, transient forms, which were not observed in the NMR spectra. In this network there is a low isomerization barrier between the *cis-* and *trans-*chalcone forms. Thus, at low pH values the reddish flavylium cationic form (*λ*_max_ at 540 nm) is the only form occurring in considerable amounts, while at more moderately acidic and at neutral pH values the yellow *trans-*chalcone form (*λ*_max_ at 420 nm) is increasing with pH to be the most dominant form. The group of Pina [10] has previously reported a similar network with similar colours for the synthetic compound 2-(4-hydroxystyryl)-1-naphthopyrylium. Here they reported that a photochromic system with good switching colours from yellow to red (or from orange to red depending on pH) based on this network could be designed. Another interesting paper from this group has also reported a similar natural photochromic system based on dracoflavylium [21]. However, this compound does not have the conjugation of the D- and E-rings of compound **1**, which gives less colour (*λ*_max_ at 374 nm) and less occurrence of the *trans*-chalcone form compared to **1**. The occurrence of the *trans*-chalcone form was in this paper improved by stabilization of the *trans*-chalcone form in the presence of β-cyclodextrin. The common anthocyanins (but the 3-deoxyanthocyanins) have similar colours as **1** for their flavylium cationic and *trans*-chalcone forms. However, opposite to the network of **1**, these natural anthocyanins have a high isomerization barrier between their *cis-* and *trans-*chalcone forms and turn into colourless hemiketal forms under moderately acidic to neutral conditions. However, photochemistry of the colourless hemiketal forms of common anthocyanins have been shown to have a potential role in plant protection from UV-B radiation [22]. The stable, reversible two-component colour system involving the reddish flavylium cation and the yellow *trans*-chalcone forms of **1** might thus be of advantage compared to previously reported multistate systems of natural anthocyanins.

## 4. Materials and Methods 

### 4.1. Isolation of Sphagnorubin C (***1***)

Sphagnorubins are natural pigments tightly bound to the cell walls of peat moss (*Sphagnum* sp.) [23], which makes them quite difficult to isolate. Additionally, in contrast to most anthocyanins, they are not soluble in water, and poorly soluble in pure methanol.

Brightly red coloured peat moss (8 kg wet wt.) was collected in the western part of Norway (60.2744 N, 5.4974 E), air dried and homogenised using a blender. The dried plant material was extracted 4 times for 24 h in 8 L 0.5% TFA in methanol (*v*/*v*). The combined extracts were concentrated to a low volume under reduced pressure. A low volume of 0.5% TFA in water (*v*/*v*) was added and the remaining methanol was removed under reduced pressure. The extract was partitioned against ethyl acetate, where three layers formed: water on the bottom, a thick layer of precipitate containing most of the Sphagnorubins in the middle and ethyl acetate on the top. The water and middle layer were removed and partitioned six more times against ethyl acetate. The purified extract was evaporated to dryness, recovered using a minimal volume of 0.5% TFA in methanol (*v*/*v*) and applied to a column of Amberlite XAD-7 resin (Supelco, Bellefonte, USA). Most of the pigments were eluted as a precipitate using 0.5% TFA in water (*v*/*v*), the eluate was evaporated to dryness under reduced pressure. Sphagnorubin C (**1**) was isolated from the extract using semi-preparative high-performance liquid chromatography (HPLC), which produced pure pigment suitable for 1D and 2D NMR spectroscopy and visible absorption spectroscopy. 

### 4.2. Samples for Analysis

Each injection on the semi-preparative HPLC system yielded about 0.15 mg of pure **1**. One batch was used to produce sample A, which was dissolved in 5% *d*-TFA in *d*-DMSO for analysis on NMR. Another batch was used to produce sample B, which was dissolved in pure *d*-DMSO for analysis on NMR. A third batch was dissolved in 10 mL 0.5% TFA in methanol and aliquoted into samples appropriate for visible absorption spectroscopy. Each aliquot was evaporated to dryness under a stream of nitrogen. These aliquots were used to produce samples C, D, and F_1_–F_4_. Sample C was dissolved in 0.5% TFA in DMSO and sample D was dissolved in pure DMSO. Sample F_1_ was identical to sample D, while samples F_2_–F_4_ was prepared by adding respectively one, two and three purification steps in addition to those used for preparation of F_1_. Each of these purification steps included adding pure methanol to pigment sample followed by evaporation to dryness under a stream of nitrogen. Samples F_3_ and F_4_ gave identical results.

### 4.3. Semi-Preparative High-Performance Liquid Chromatography (HPLC)

Semi-preparative HPLC were performed using a Gilson 321 pump (Gilson, Inc., Middleton, WI, USA) equipped with an UltiMate 3000 variable wavelength detector (Thermo Fisher Scientific, Waltham, MA, USA) set at 540 nm, a 25 × 2.2 cm (10 µm particle size) Econosphere C18 column (Grace, Columbia, MD, USA). The mobile phases were A, 1% TFA in water (*v*/*v*), and B, 1% TFA in methanol (*v*/*v*), used at a flow of 15 mL/min. A solvent elution profile consisted of initial conditions of 30% B and the following isocratic and gradient elution: 0–14 min gradient to 40% B, 14–20 min gradient to 50% B, 20–30 min isocratic, 30–32 min gradient to 80% B, 32–38 min isocratic, and a finally 38–41 min gradient to 30% B. Prior to injections, samples were filtered through a 0.45 µm filter.

### 4.4. Analytical HPLC

Analytical HPLC was used to assess the purity of the extract during each step of the isolation procedure. The instrument used was an HP Agilent 1200 analytical HPLC system (HP/Agilent, Santa Clara, USA) equipped with a diode array detector at 520 nm and 280 nm, and a 250 × 25 mm (4.5 µm particle size) Ascentis RP-Amide column (Supelco, Bellefonte, USA. The mobile phases were A, 1% TFA in water (*v*/*v*), and B, 1% TFA in acetonitrile (*v*/*v*), used at a flow of 1 mL/min. A solvent elution profile consisted of initial conditions of 30% B and the following isocratic and gradient elution: 0–7 min gradient to 40% B, 7–10 min gradient to 50% B, 10–15 min isocratic, 15–17 min gradient to 80% B, 17–22 min isocratic, and 22–25 min gradient to 30%. Injections were 20 µL aliquots injected by an auto-sampler. Prior to injections, samples were filtered through a 0.45 µm filter.

### 4.5. Nuclear Magnetic Resonance (NMR) Spectroscopy

The NMR experiments were obtained using a Bruker Ultrashield Plus AV-600 MHz (Bruker, Billerica, MA, USA) at 600.13 and 150.92 MHz for ^1^H and ^13^C spectra respectively, recorded at 21 °C. Deuterated dimethyl sulfoxide (*d*-DMSO) and deuterated trifluoroacetic acid (*d*-TFA) were used as solvents. ^1^H and ^13^C signals of the spectra were calibrated using the *d*-DMSO residual signal at 2.50 ppm and 39.51 ppm, respectively.

### 4.6. Mass Spectrometry

Purified samples of both the flavylium cation and the *trans*-chalcone of **1** were analysed by high-resolution electron spray ionisation mass spectrometry (HR-ESI-MS). The spectra were recorded using a JEOL AccuTOF instrument (JEOL USA, Inc., Peabody, MA, USA). See supplementary Figures S7 and S8 for details about the individual MS spectra.

### 4.7. Visible Absorption Spectroscopy

A HunterLab UltraScan PRO spectrophotometer (Hunter Associates Laboratory, Inc., Reston, VA, USA) was used to record visible absorption spectra. Pure solvents were used as background and extracted from each spectrum. The instrument was calibrated every 24 h. During 24 h of interval sampling, the cuvettes were covered with parafilm. The data from the instrument were imported to Microsoft Excel for data analysis.

## Figures and Tables

**Table 1:** Sphagnorubin C (**1**) ^1^H and ^13^C-NMR data of the flavylium cation form in 5% *d*-TFA in *d*-DMSO (*v*/*v*) and the *trans*-chalcone form (after 24 h of equilibration) in pure *d*-DMSO.

Position	Position	^1^H *δ* (ppm) *J* (Hz)	^1^H *δ* (ppm) *J* (Hz)	^13^C *δ* (ppm)	^13^C *δ* (ppm)
flav	*t*-chal	flav	*t*-chal	flav	*t*-chal
2	CO			168.98	187.78
3	α	8.74 *d* 9.0	7.97 *d* 15.4	113.28	123.38
4	β	10.04 *d* 9.1	8.34 *d* 15.4	148.17	136.88
4a	1			117.81	108.77
4b	10a			129.43	132.83
5	10	8.72 *d* 9.0	7.87 *d* 9.2	117.89	118.60
6	9	8.20 *d* 8.9	7.67 *d* 9.2	131.49	128.25
6a	8a			129.27	125.74
7	8	7.47 *s*	7.18 *s*	111.95	111.62
8	7			147.68	145.02
9	6			147.99	146.10
10	5	9.05 *s*	8.89 *s*	112.43	112.61
10a	4b			123.77	124.12
10b	4a			119.70	114.23
11	4			168.68	159.91
12	3	8.13 *s*	6.90 *s*	97.68	98.60
12a	2			158.35	156.38
1’	1’			120.15	130.09
2’	2’	8.09 *d* 2.2	7.56 *d* 2.0	112.18	111.35
3’	3’			148.92	147.69
4’	4’			155.39	151.31
5’	5’	7.16 *d* 8.6	6.93 *d* 8.2	116.83	115.08
6’	6’	8.26 *dd* 2.2, 8.6	7.59 *dd* 2.0, 8.2	125.46	122.84
MeO-C3’	MeO-C3’	4.03 *s*	3.87 *s*	56.49	55.56
MeO-C11	MeO-C4	4.44 *s*	4.08 *s*	58.15	55.61
*s*: singlet, *d*: doublet, *dd*: doublet of doublets					

## Data Availability

The data presented in this study are available on request from the corresponding author.

## Supplementary Materials

Supplementary **Table S1**: Peonidin 3-*O*-glucoside (**2**) ^1^H-NMR data. Supplementary Figure S1: ^1^H-NMR spectrum of the flavylium cation form of Sphagnorubin C (**1**) dissolved in 5% *d*-TFA in *d*-DMSO. Supplementary Figure S2: Heteronuclear Multiple Bond Correlation (HMBC) NMR spectrum of the flavylium cation form of Sphagnorubin C (**1**) dissolved in 5% *d*-TFA in *d*-DMSO. Supplementary Figure S3: Heteronuclear Single Quantum Coherence (HSQC) NMR spectrum of the flavylium cation form of Sphagnorubin C (**1**) dissolved in 5% *d*-TFA in *d*-DMSO. Supplementary Figure S4: Double-quantum filtered correlation spectroscopy (DQF-COSY) NMR spectrum of the flavylium cation form of Sphagnorubin C (**1**) dissolved in 5% *d*-TFA in *d*-DMSO. Supplementary Figure S5: DQF-COSY NMR spectrum of the flavylium cation and *trans*-chalcone forms of Sphagnorubin C (**1**) dissolved in pure *d*-DMSO and equilibrated for 24 h before recording. Supplementary Figure S6: HMBC NMR spectrum of the flavylium cation and *trans*-chalcone forms of Sphagnorubin C (**1**) dissolved in pure *d*-DMSO and equilibrated for 24 h before recording. Supplementary Figure S7: Mass spectrometry spectrum of the flavylium cation form of Sphagnorubin C (**1),** and instrument settings used for the recording. Supplementary Figure S8: Mass spectrometry spectrum of the *trans*-chalcone form of Sphagnorubin C (**1),** and instrument settings used for the recording.
