# Supplementary material for: Characterization of a Natural, Stable, Reversible and Colourful Anthocyanidin Network from Sphagnum Moss Based Mainly on the Yellow Trans-Chalcone and Red Flavylium Cation Forms
Source: Molecules. 2021 Jan 29;26(3):709. doi: 10.3390/molecules26030709 (PMC7866509; doi:10.3390/molecules26030709)
Supplement: Supplementary file 1 [file molecules-26-00709-s001.zip › Table S1 NMR shift values pn3glc.docx]

**Table S1**: Peonidin 3-glucoside (**2**) ^1^H NMR data of the flavylium cation form in 5 % *d*-TFA in *d*-DMSO (v/v) and the two hemiketal forms a and b in pure *d*-DMSO recorded after 24 hours of equilibration

| Position | ^1^H *δ* (ppm) J (Hz) | ^1^H *δ* (ppm) J (Hz) | ^1^H *δ* (ppm) J (Hz) |
| --- | --- | --- | --- |
| **flav** | **flavylium** | **hemiketal a** | **hemiketal b** |
| 4 | 8.94 *s* | 6.35 *s* | 6.16 *s* |
| 6 | 6.72 *d* 2.0 | 5.90 *m* | 5.90 *m* |
| 8 | 7.04 *d* 2.0 | 5.65 *m* | 5.65 *m* |
| 2' | 8.18 *d* 2.2 | 7.15 *d* 1.9 | 7.01 *d* 2.0 |
| 5' | 7.09 *d* 8.6 | 6.70 *d* 8.0 | 6.69 *d* 8.0 |
| 6' | 8.28 *dd* 8.6, 2.2 | 6.75 *dd* 8.0, 1.9 | 6.85 *dd* 8.2, 2.0 |
| 4'-OMe | 3.92 *s* | 3.74 *s* | 3.72 *s* |
| 1'' | 5.38 *d* 7.8 | 4.68 *d* 8.1 | 4.99 *d* 8.1 |
| *s*: singlet, *d*: doublet, *dd*: doublet of doublets, *m*: multiplet | | | |
