# Supplementary material for: Characterization of a Natural, Stable, Reversible and Colourful Anthocyanidin Network from Sphagnum Moss Based Mainly on the Yellow Trans-Chalcone and Red Flavylium Cation Forms
Source: Molecules. 2021 Jan 29;26(3):709. doi: 10.3390/molecules26030709 (PMC7866509; doi:10.3390/molecules26030709)
Supplement: Supplementary file 1 [file molecules-26-00709-s001.zip › Figure S8 ESI- HR-MS trans-chalcone form.pdf]

Experiment
Needle Volt: -2200[V]
Ring Lens Volt: -10[V]
Ion Guide Bias Volt: -30[V]
Acquired m/z Range: 100.00..1000.00
Spec. Record Interval: 0.60[s]

Orifice1 Volt: -23V
Desolvating Chamber Temp: 250[°C]
Pusher Bias Volt: 0.80[V]
Data Acquisition Interval: 1[ns]
Wait Time: 0.023[s]

Ionization Mode: ESI-
Orifice2 Volt: -6[V]
Ion Guide RF Volt: 1600V
Detector Volt: 2350[V]
Flight Repetition Interval: 59[µs]
Detector Volt: 2350[V]

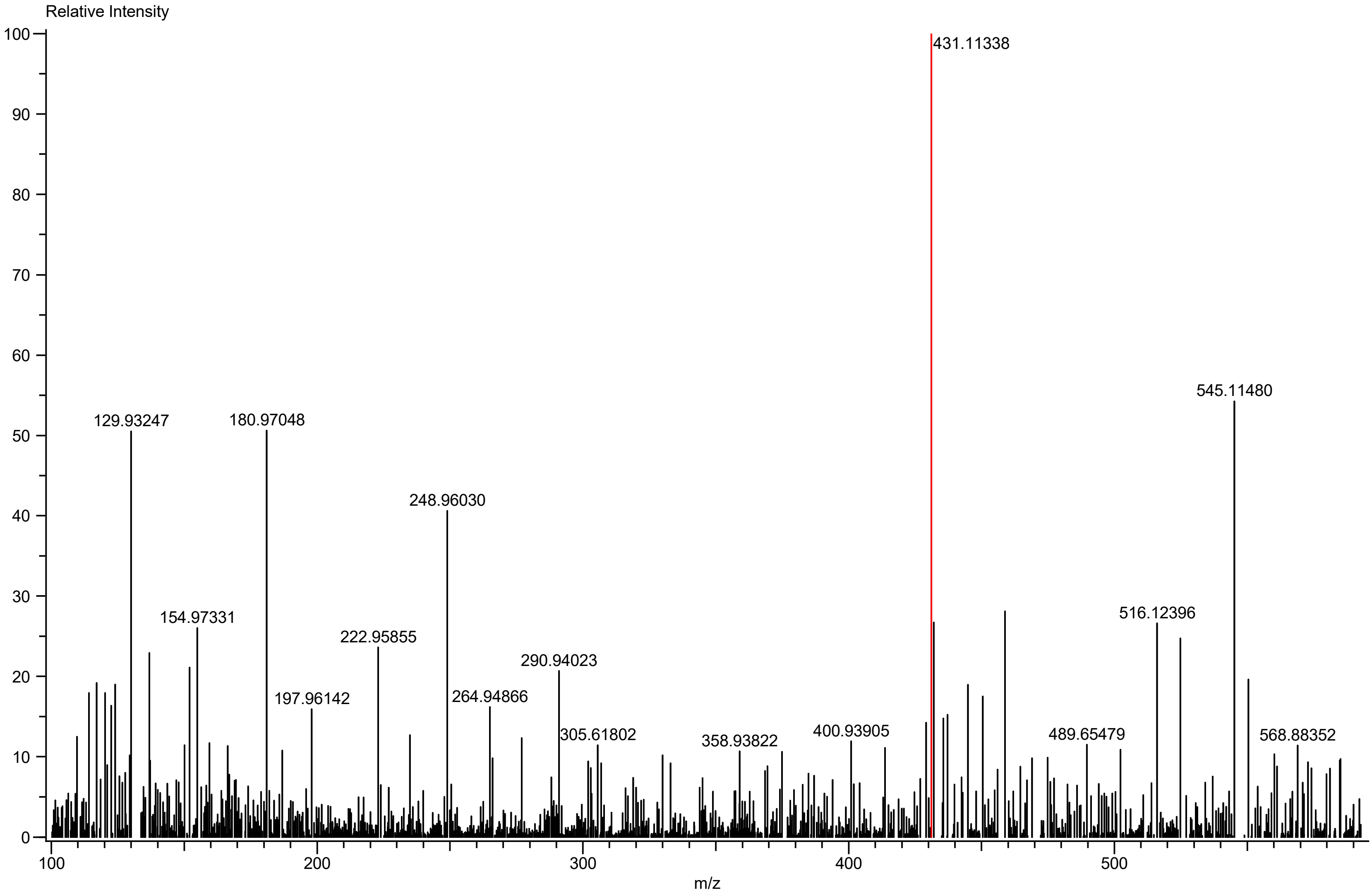

History:Determine m/z[Peak Detect[Centroid,50,Area];Correct Base[5.0%]];Average(MS[1] 0.494..0.519)-1.0*Avera...

Created by:Accutof

Charge number:1

Tolerance:10.00(mmu)

Unsaturation Number:-1.5 .. 40.0 (Fraction:Both)

Element:^{12}C:0 .. 100, ^{1}H:0 .. 200, ^{16}O:0 .. 10

Mass	Intensity	Calc. Mass	Mass Difference (mmu)	Possible Formula	^{12}C	^{1}H	^{16}O	Unsaturation Number
431.11338	26125.29	431.11308	0.31	$^{12}C_{25}{}^{1}H_{19}{}^{16}O_{7}$	25	19	7	16.5
		431.10720	6.18	$^{12}C_{32}{}^{1}H_{15}{}^{16}O_{2}$	32	15	2	25.5
