# Supplementary figures and images for: Characterization of a Natural, Stable, Reversible and Colourful Anthocyanidin Network from Sphagnum Moss Based Mainly on the Yellow Trans-Chalcone and Red Flavylium Cation Forms
Source: Molecules. 2021 Jan 29;26(3):709. doi: 10.3390/molecules26030709 (PMC7866509; doi:10.3390/molecules26030709)

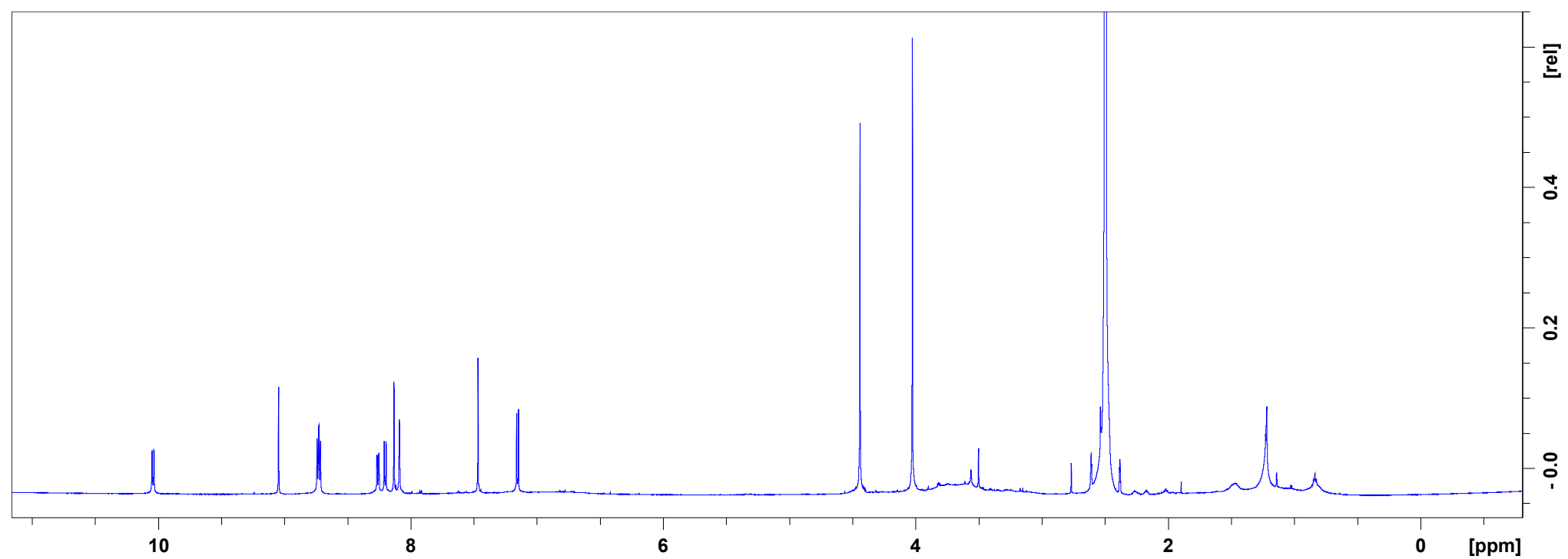

Supplement: Supplementary file 1 [file molecules-26-00709-s001.zip › Figure S1 1H NMR SPHC flavylium.pdf]

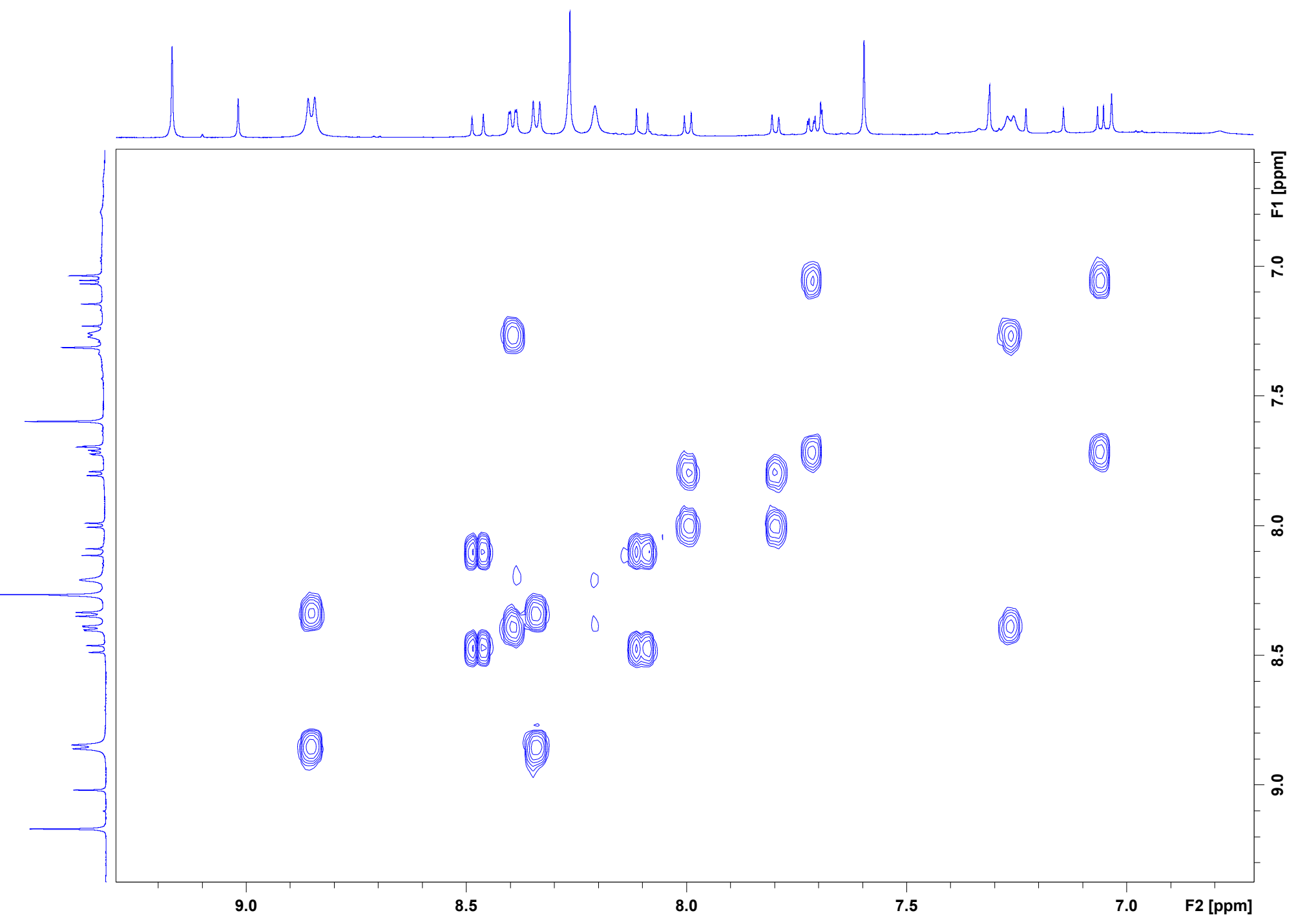

Supplement: Supplementary file 1 [file molecules-26-00709-s001.zip › Figure S5 DQF-COSY d-DMSO.pdf]

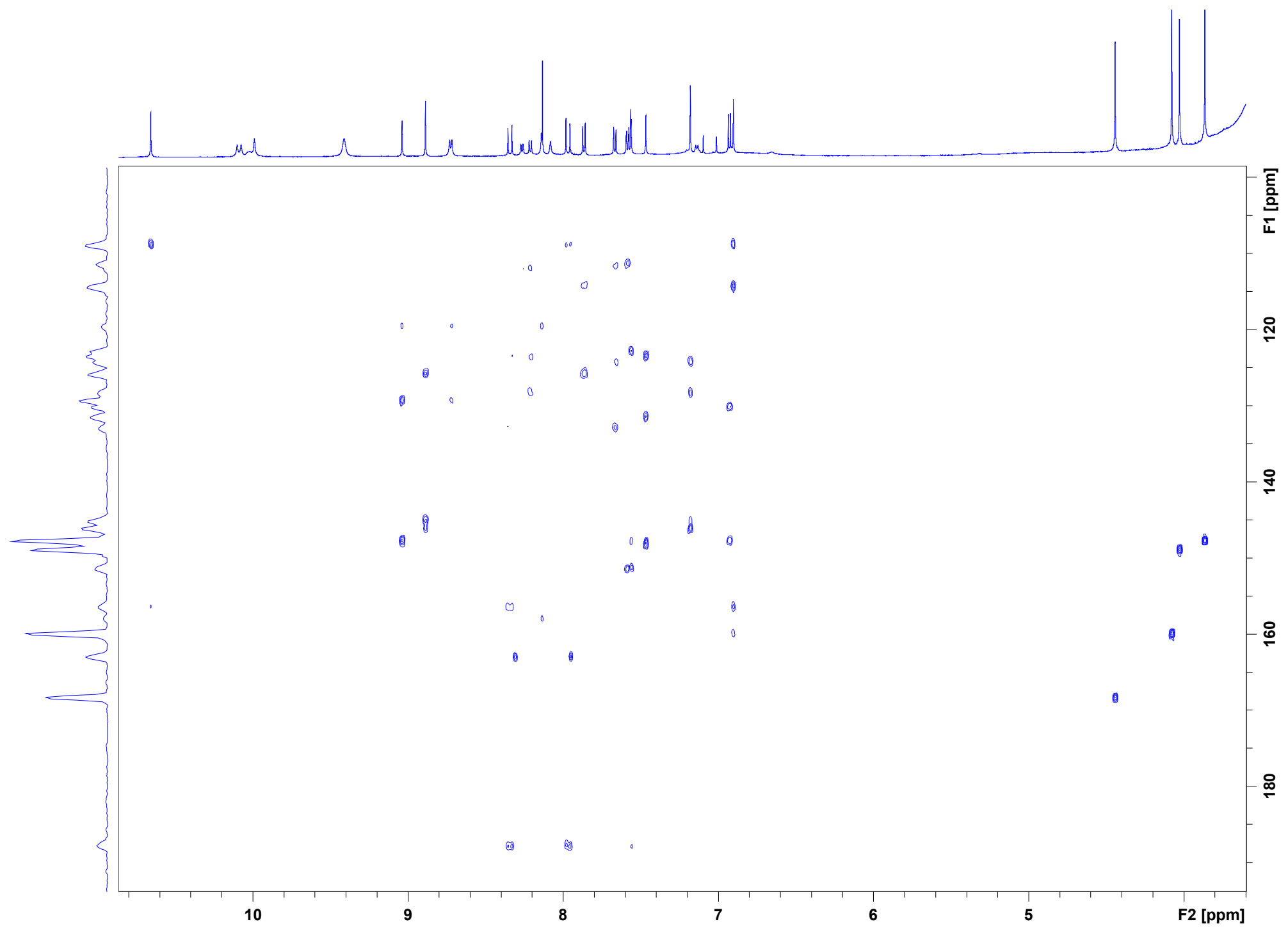

Supplement: Supplementary file 1 [file molecules-26-00709-s001.zip › Figure S6 HMBC d-DMSO.pdf]

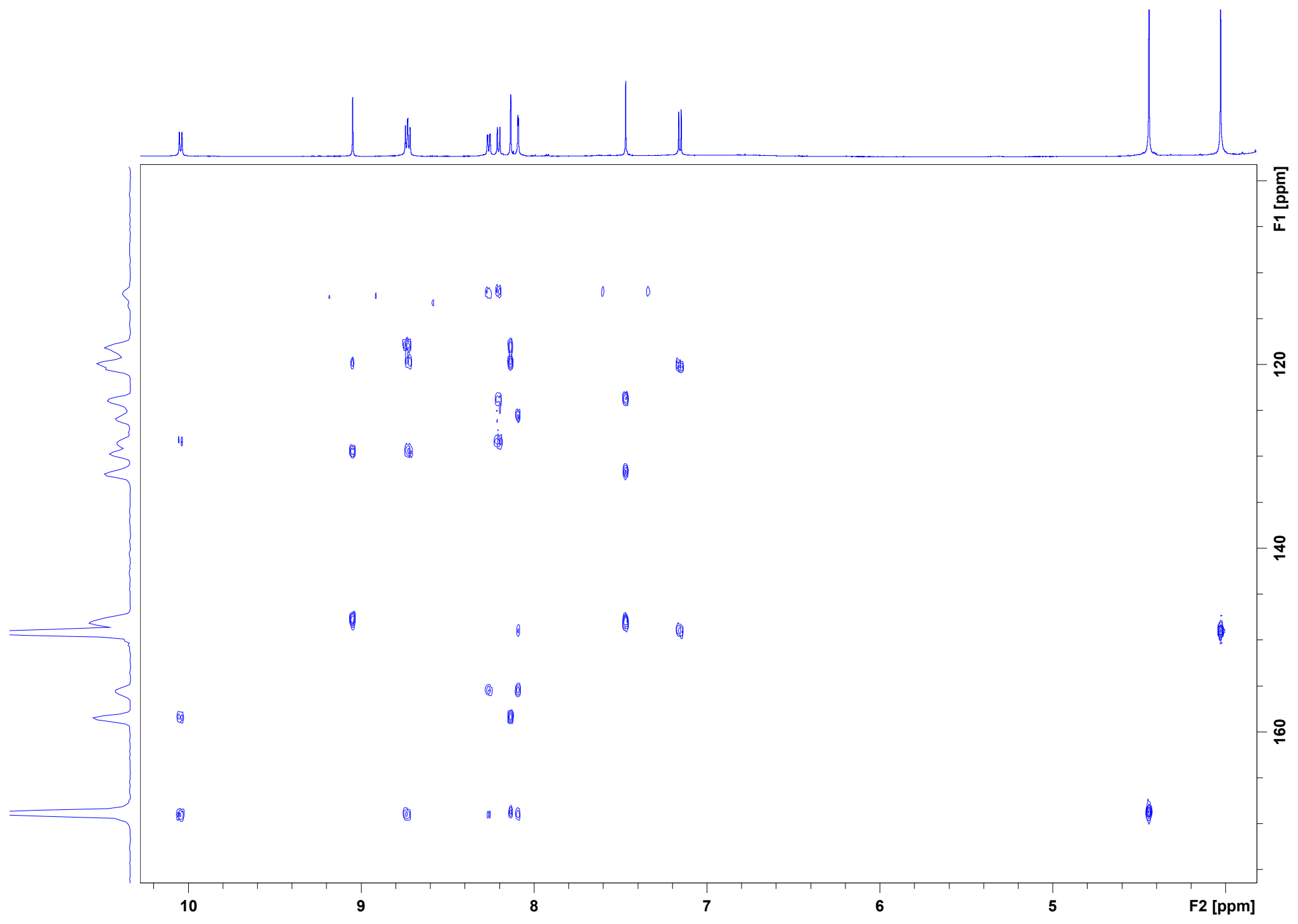

Supplement: Supplementary file 1 [file molecules-26-00709-s001.zip › Figure S2 HMBC in acidic DMSO.pdf]

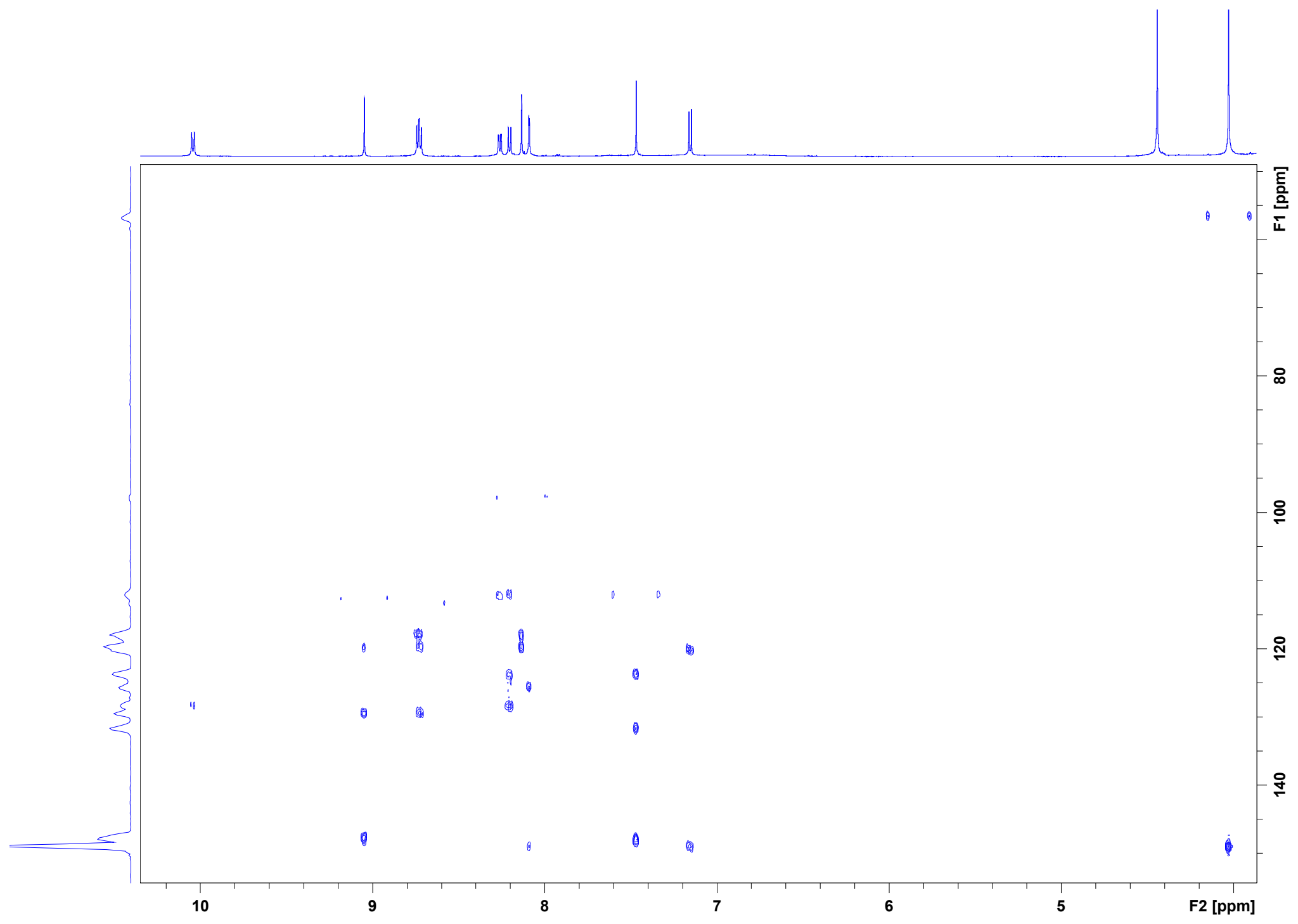

Supplement: Supplementary file 1 [file molecules-26-00709-s001.zip › Figure S3 HSQC in acidic DMSO.pdf]

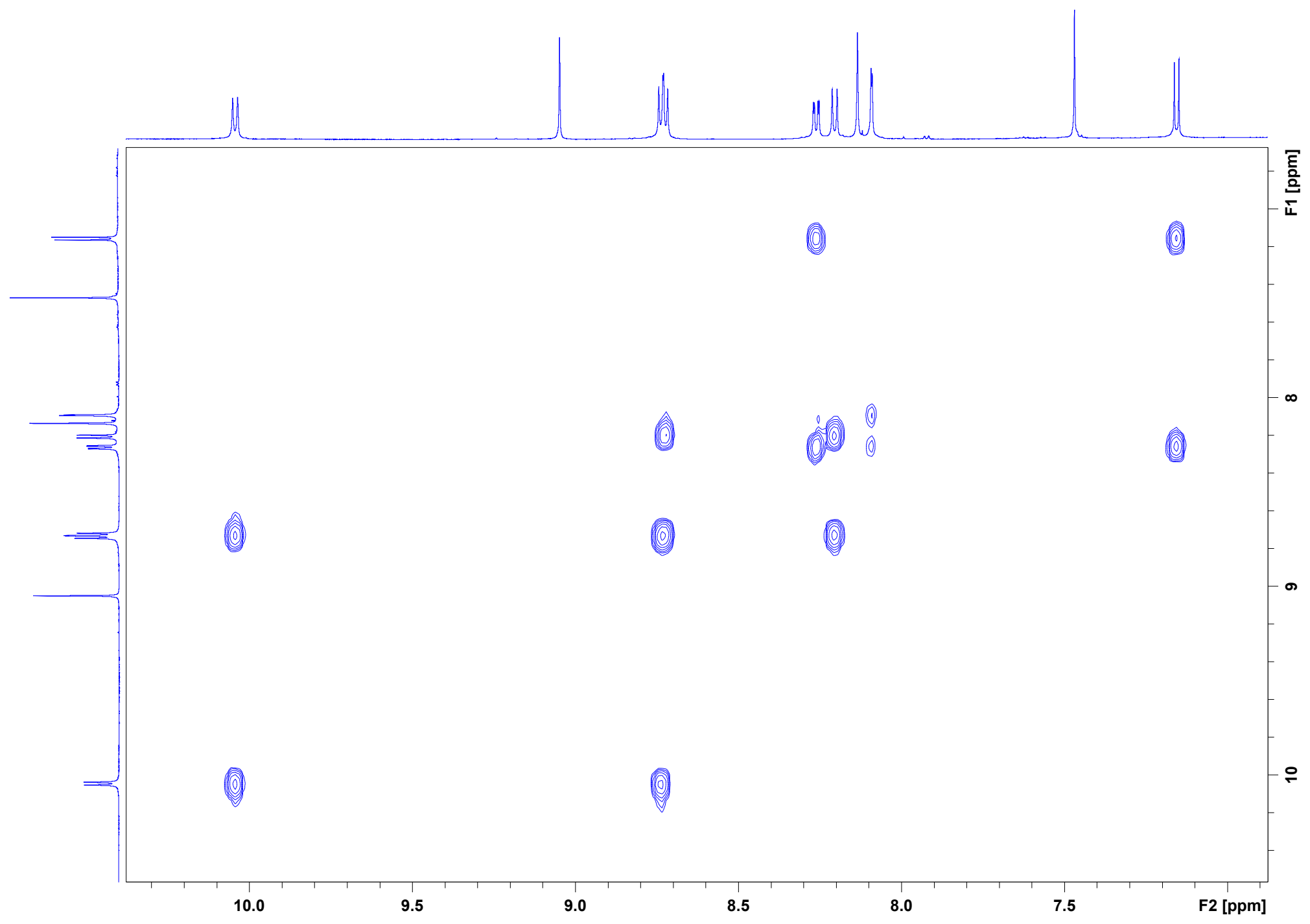

Supplement: Supplementary file 1 [file molecules-26-00709-s001.zip › Figure S4 DQF-COSY in acidic DMSO.pdf]
